# Indicators to assess preservice teachers’ digital competence in security: A systematic review

**DOI:** 10.1007/s10639-022-10978-w

**Published:** 2022-03-09

**Authors:** Norma Torres-Hernández, María-Jesús Gallego-Arrufat

**Affiliations:** Faculty of Education , University of Granada, Granada, Spain

**Keywords:** Digital competence, Internet security, Safety education, Preservice teachers, Teacher education, Higher education

## Abstract

The goal of this review is to analyse the state of inquiry in the field of digital competence in security in initial teacher education, via indicators to assess preservice teachers’ digital competence in security, in order to help find opportunities to improve their competence level. Following the parameters defined in the PRISMA declaration, the review uses a bibliographic research methodology to explore the WoS, Scopus and ERIC databases. After a search identifying a sample of 31 scholarly articles published between 2010 and 2021, we analyse the information obtained using descriptive statistics and content analysis. The results show a predominance of empirical research in the European context. These studies are quantitative and tend to use questionnaires. Our conclusion proposes the need to train preservice teachers in data protection and privacy, searching for and using Internet images with authorship screening, use of open software programs, and respect for online communication norms, as well as ethical and responsible technology use. All of these issues are implicitly and transversally linked to the area of digital competence in security.

## Introduction

Digital competence is a central goal in educating twenty-first-century citizens. In higher education in the past decade, various initiatives and programs—by the United Nations Educational, Scientific and Cultural Organization (UNESCO), the Organization for Economic Co-operation and Development (OECD), the European Commission (EU), the International Society for Technology in Education (ISTE), and the British Educational Communications and Technology Agency (BECTA)—have proposed standards, indicators and recommendations in reports and studies of educators’ digital competence. These efforts show clear interest in generating better competence profiles for technology use in education.

Information and Communication Technologies (ICTs) and Internet use have brought significant advances and benefits to society, but we also know that their use brings multiple risks, due to lack of information and training about security in digital environments. In the educational environment, it is important for teachers not only to be aware of the problems and risks, but also to know how to identify and prevent them (Kritzinger, 2017). Society is concerned about these risks, especially about privacy and education to foster responsible use of Internet (Chou & Peng, 2011). Organizations and institutions thus seek to build a climate of trust to mitigate or prevent the effects of problems related to digital security (e-safety), especially as oriented to vulnerable groups, and to promote actions that orient, inform and safeguard digital security and knowledge of digital rights.

Responding to digital trust in the educational environment requires professors who are more digitally competent (Cabezas et al., 2017; Fulgence, 2020; Instefjord & Munthe, 2017; INTEF, 2017, Šimandl & Vaníček, 2017) in knowledge (content and pedagogy), skills (social and technical) and attitudes—especially as these relate to digital security.

Although we know that education systems currently recognize the importance of training teachers to master ICTs, and especially ICT security, we lack knowledge of what basic capabilities to require in initial training and how to assess them (Cózar & Roblizo, 2014; Cebrián-de-la-Serna et al., 2014).

Given this topic’s importance to education and the value of contributing to digital security, this review covers the significant development in the past decade of the topic of initial training. It synthesizes this literature and provides the first exhaustive analysis to date.

The goal of the systematic review is to identify and systematize scholarly production on this topic, focusing on indicators to assess the area of digital competence in security within the European Digital Competence Framework for Educators (DigCompEdu) (Redecker, 2017).

## Conceptual framework

### Digital competence in education

Digital competence is fundamental to preservice teachers’ education. In addition to contributing to improving their professionalization (Pozos-Pérez & Tejada-Fernández, 2018), it is necessary to face the new challenges of digital society successfully. In Europe, the strategy of acquiring digital competences insists on the importance of achieving a minimal competence level at different stages of education. It also insists that improvement requires the involvement and lifelong assessment of teachers (European Commission, 2018). Definitions of digital competence conceptualize people’s capabilities for technology use as including appropriation, understanding of ethical questions and critical use (Ilomäki et al., 2016). Based on the foregoing, we believe that digital competence can help to orient learning in a digital society that is undergoing constant transformation (Hall et al., 2014), while simultaneously encouraging the critical, responsible, creative technology use. Digital awareness is thus fundamental for training in education processes and participation in twenty-first-century society (Napal et al., 2018). According to Watson (2006), schools must engage in debates on questions of responsible technology use and the problems involved, and instruction on these topics is key for schools, teachers and families. Personal and collective responsibility must be developed through debate and understanding that the digital world also has social norms that must be observed and respected; only in this way can we interact and live more securely.

### Digital competence for educators

Digital competence for educators is the set of personal characteristics, knowledge, skills and attitudes needed to act effectively in diverse contexts that permit teaching and learning with didactic, pedagogical and methodological criteria and full critical, moral and ethical consciousness (Tigelaar et al., 2004). According to DigCompEdu (Redecker, 2017), this framework is composed of five areas, subdivided into 21 competences. Security is a cross-cutting field but also has its own character. It is divided into four competences, each with different indicators on protection of devices, protection of personal data and digital identity, protection of the environment and protection of health. Related to this area are the concepts of digital culture, digital competence, digital security, problems and risks on Internet (Hutson et al., 2018). This competence has practical, transversal use in all learning activities that preservice teachers perform during their education. It is necessary to drive, understand and deepen knowledge in use and to assume a more responsible attitude (Cózar-Gutiérrez et al., 2016). The current educational scenario caused by the COVID-19 pandemic has shown the importance of digital teaching competences as the use of technology has become essential in all educational areas (Meinokat & Wagner, 2021). This scenario has led to the putting into practice of skills related to the search for, selection and use of information; and types of organization, use and communication among professors, parents and alumni (Tomczyk & Walker, 2021) that involve secure, ethical, legal responsible Internet use.

### Security in digital education

Digital security, *Internet Security* or *Internet Safety* is a cross-cutting area that involves using a set of knowledge, skills and attitudes to browse the Internet or use technological devices securely. For Chou and Peng (2011), e-Safety includes privacy, integrity and efficiency of Internet, defining the term as protection of users’ information and communication against the problems generated by ICT use (Barrow & Heywood-Everett, 2006). In the educational environment, the idea of security has two aspects that are complementary and also belong to other competence areas. The first is linked to knowledge for security, which seeks to promote a healthy, secure environment for handling equipment with appropriate knowledge of protection and security to protect information and communication with other users (Le et al., 2015; Barrow & Heywood-Everett, 2006; Anderson, 2003). The second is related to education for digital security, which aims to give teachers and students the knowledge and skills needed to ensure security. Both views are necessary for creating digitally secure, critical and conscious citizens (Pham et al., 2019).

### Training in security and ethical, secure, responsible technology use

For teachers exercising digital competence, it is not enough to know how to make or use technology efficiently, as the effects of the pandemic have clearly shown. In addition to instrumental skills in handling technology, teachers need initial training in suitable, critical, reflexive and ethical use of technologies (Novella-García & Cloquell-Lozano, 2021). As Fernández-de-la-Iglesia et al. (2020) affirm, such training would also enable teachers to acquire better levels of digital competence and to understand and apply ethical criteria for using technology properly and responsibly. Watson (2006) observes that these social and ethical aspects of technology use are an area of significant interest but have received little attention in school agendas. This observation is generally supported by the fact that teacher training in digital competence usually focuses on improving skills in handling artifacts (information literacy). Yet such training alone does not enable teachers to solve social, psychological or educational problems that arise in the classroom. The shortcomings are even greater if the initial teacher training plans in study programs do not include much material on the ethical dimension (Novella-García & Cloquell-Lozano, 2021).

Current educational focus on security includes digital education programs and programs for prevention that disseminate content. Their goal is to enable teaching to promote, model and train students as digitally responsible citizens (Torres-Hernández et al., 2019) who care for and protect their privacy, identity and digital footprint while also caring for their health and protecting the environment (Castillejos et al., 2016). Studies such as Faherty et al. (2019) show interest in guaranteeing students’ security as an emerging question to which education must attend. Despite the incorporation of some training programs in this direction, however, study programs lack assessment materials to confirm the efficacy of their practice (Jones et al., 2014). Nor do they know how schools have contributed to improving digital competence and creating better conditions for safer browsing in teaching and learning. Some initial training studies of digital competences in security have concluded that the quality of the initial training of teachers is deficient (Björk & Hatlevik, 2018; Gilant, 2016; Govender & Skea, 2015; Shin, 2015), leading to proposals to include security in universities’ educational agendas at the different levels of instruction (Atkinson, 2009; Avello et al., 2014; Ocaña-Fernández et al., 2020).

To respond to the problems that arise related to technology use and security breaches, developing actions to integrate curricula for training in and improvement of digital competences in this area requires not only political willingness but also a long-term view of training responsible, secure citizens in this century—a goal that presents a tremendous didactic, pedagogical and technological challenge (Ranjbari et al., 2020; Nganji, 2018) in which teachers and schools must know a lot and do even more.

## Method

This review is guided by the following questions:

- RQ1. What combinations of keywords are often used in educational research on digital security?
- RQ2. What are the methodological characteristics of studies in education research on digital security?
- RQ3. What competence levels do preservice teachers have in research on digital security?
- RQ4. What indicators are found in studies of preservice teachers’ digital competences that enable assessment of digital security?
- RQ5. What opportunities for improvement in teachers’ digital competence in security appear in the studies?

**Fig. 1:**
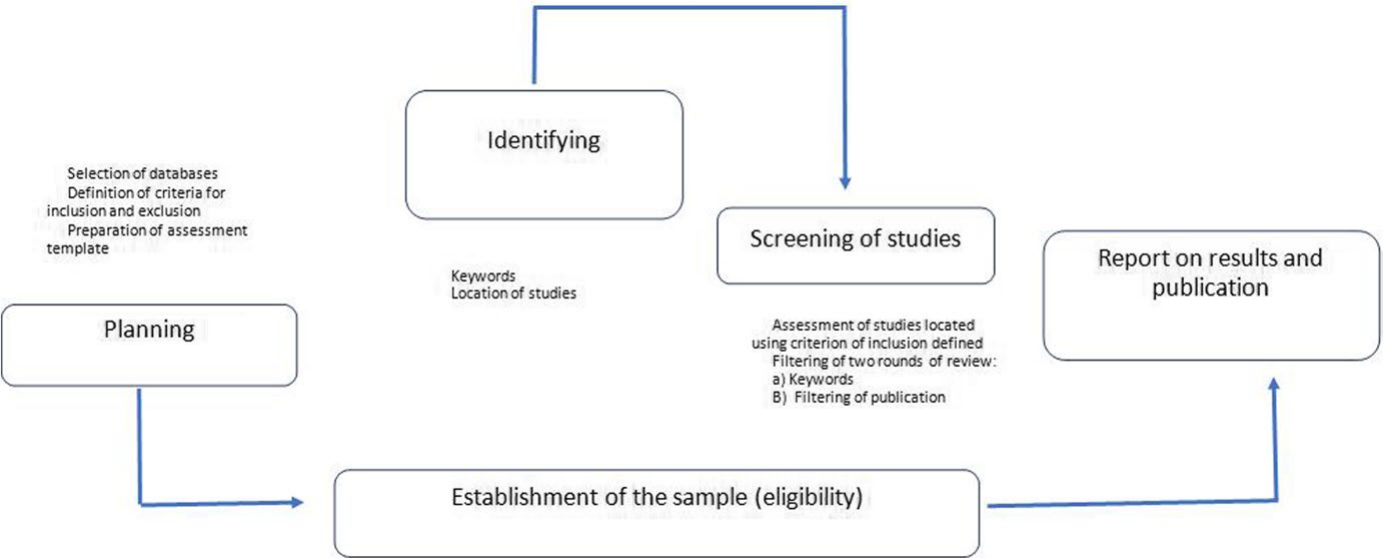
Stages of the systematic review (Kitchenham, 2004; Kitchenham & Charters, 2007)

We use the research method appropriate for systematic literature reviews (Fink, 2019), following the directives of the *Preferred Reporting Items for Systematic Reviews and Meta-Analyses* (PRISMA) Declaration. Adopted by the American Psychological Association (APA), this report is an important guide providing detailed explanation of methodological issues that enable us to assess the reliability and applicability of results in this type of study in the field of education. The tasks of planning, identification, screening and reporting illustrated in Fig. 1 follow the procedures for conducting systematic literature reviews outlined in Kitchenham (2004) and Kitchenham and Charters (2007). These tools enable us to follow a systematic method to locate, select and assess critically the relevant research and apply a protocol in this observational, retrospective, secondary study.

### Criteria of inclusion and exclusion

The general criterion of inclusion is that the publication have been found in one of the following categories in the WoS, Scopus or ERIC databases: *Education & Educational Research* (Web of Science Core Collection-SSCI), *Education* (Scopus-Social Sciences) or ERIC. The study’s criteria of inclusion (CI) were: research published 2010–2020 (CI1), studies with preservice teachers as sample or participants (CI3), English and/or Spanish language (CI4) and studies with item(s) related to any of the digital teaching competences in security (DigCompEdu, 2017) (CI5). The criteria of exclusion (CEX) were presentations at congresses, book chapters, conference papers, theses and other types of publications (CEX1), and restricted access to the documentation published (CEX2).

### Procedure

The process of identifying the sample begins with identification of articles published between 2010 and 2021 in the Web of Science (SSCI), Scopus and ERIC databases. Screening used the keywords *Digital Competencies, Teacher Digital Competence, Digital Safety, Higher Education, Teacher Training, Internet Use,* and *Evaluation*; the search used the Boolean operator AND in the combinations.

We found 617 articles, 141 of which were duplicates. Applying the criteria of inclusion/exclusion reduced this number to 476 articles. A total of 300 articles were excluded because they did not meet criteria CI3, CI4 or CI5.

**Fig. 2:**
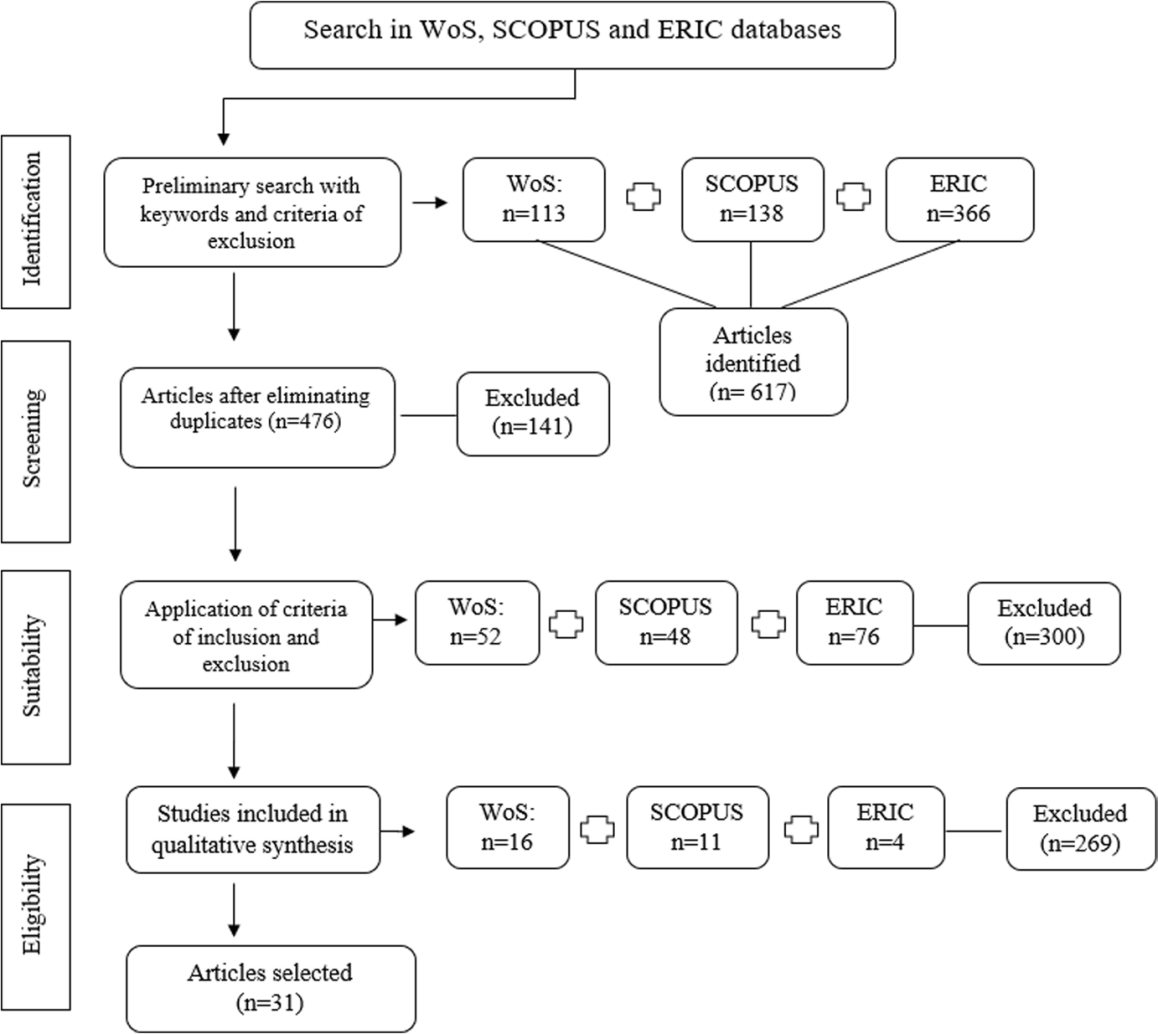
Review process, following Moher et al. (2009)

To ensure suitability and validity of the review (Bennett et al., 2005), we performed a second screening by reading the abstracts of 176 articles. In this step, we identified and chose 31 studies for more exhaustive analysis. These data screened by database are presented in the flow diagram following the process recommended in PRISMA (Fig. 2).

To systematize the results, we used Microsoft Excel to record entries on each publication with its authors, publication year, journal, location and number of citations. We also noted the following issues relevant to the analysis: key words, abstract, type of study, size of sample of participating population and sample origin, as well as the instruments used. Finally, we included a section on indicators of digital security.

Data analysis procedure: To analyse and interpret the information in this review, we used descriptive statistical techniques combined with content analysis of the studies’ keywords, abstract, results and conclusions.

The descriptive statistical analysis was first performed with Microsoft Excel. We analysed the frequency of the keywords, research methodology of the studies, countries in which they were performed and number of participants in the studies. The descriptive analysis of the sample selected provided results on frequencies and percentages by combining content analysis with the help of the reference manager Mendeley. Content analysis was performed by category using coding procedure and thematic grouping*.*

## Results

We present the results according to the research questions.

- RQ1. What combinations of keywords are often used in educational research on digital security?

The keyword combinations with the most results were “Digital Competences AND Higher Education” (30.9%), “Internet use AND Teacher Training” (29%) and “Digital Competencies AND Teacher Training” (24.4%). The combined search “Evaluation AND Digital Security”, in contrast, had the least indexed scholarly production (6%).

Analysis of keywords included in the studies showed that the most frequent combinations were *Digital Competence* and *Teacher Training*, followed by *Technology* and *E-Safety* or *Safe* (Table 1).

**Table 1:** Absolute frequencies of studies published in each database, by keywords combination

Keywords	WoS	Scopus	ERIC	Total
“Digital competencies” AND “Teacher Training”	37	43	71	151
“Digital competencies” AND “Higher Education”	49	54	88	191
“Digital Security” AND “Higher Education”	6	5	7	18
“Evaluation” AND “Safety” AND “Education”	2	3	10	15
“Evaluation” AND “Digital Security”	2	1	3	6
“Digital competencies” AND “Higher Education” AND “Teacher Training”	14	12	17	43
“Internet use” AND “Teacher Training”	3	10	166	179
“Evaluation” AND “Digital competencies” AND “Safety”	–	10	4	14
Total	113	138	366	617

Source: The authors

**Fig. 3:**
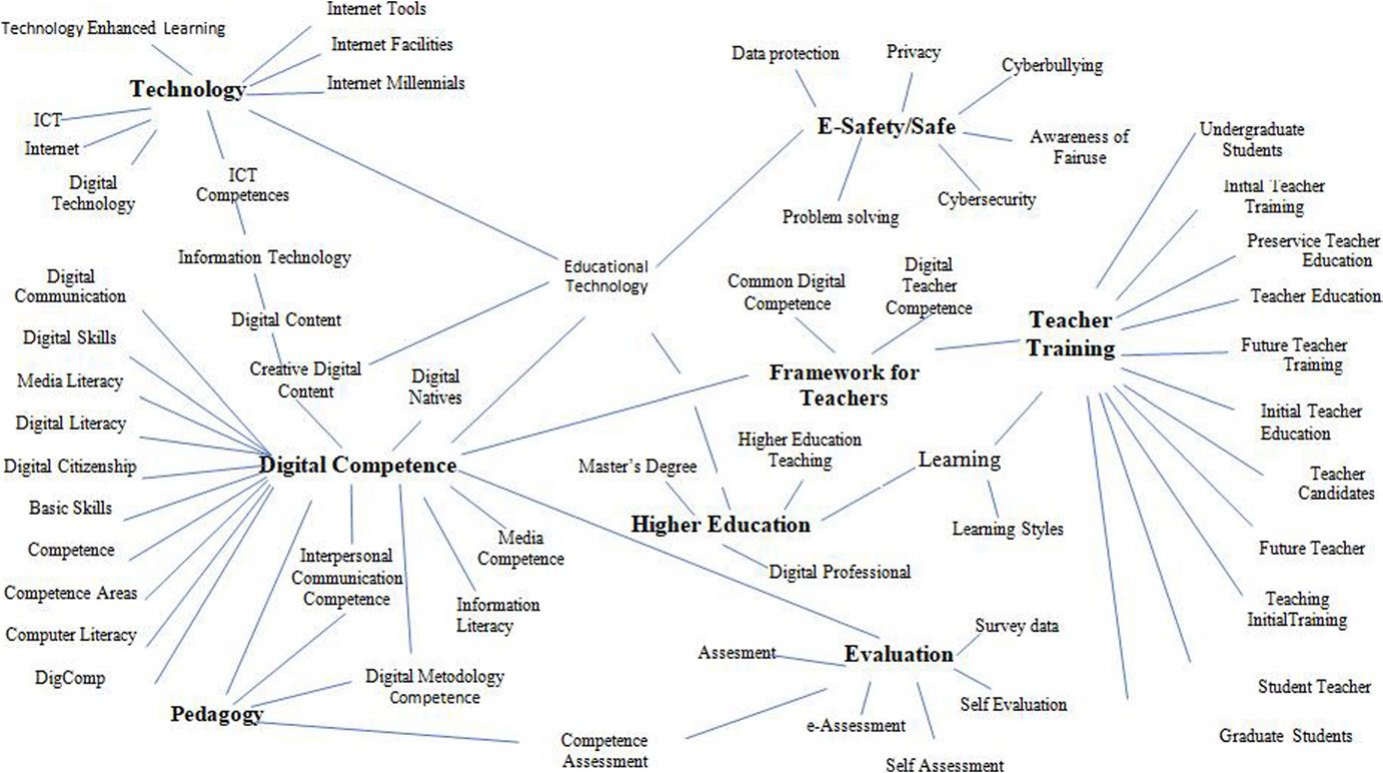
Association among keywords from the studies

In Fig. 3, the semantic fields show the following associations among the studies’ keywords: *Digital Competence* is related to 20 keywords, *Teacher Training* to 13, *Technology* to 9 and E-Safety/Safe to 7. The analysis also shows the presence of other keywords of interest for the topic of digital security and the goals of this study, such as *Evaluation* and *Higher Education* (with 6 and 4 interrelated words, respectively).

- RQ2. What are the methodological characteristics of studies in education research on digital security?

Table 2 presents the publications included in the review by number of citations (C). The table enables identification of their authors, as well as year of publication, methodology (M), description of the participants or sample (PS) and instrument used (I). The results provided enable us to show that 100% of the articles in the sample include in their methodology (in the instruments section), in their results, or in their conclusions one or several indicators for assessment of digital teaching competence in security.

**Table 2:** Methodology of the studies included in the review

Author	M	PS	I*	C
Shin (2015)	C	60	TP and LP	51
Prendes-Espinosa et al. (2010)	Q	751	Q	47
Zempoalteca et al. (2017)	Q	361	Q	29
Lázaro-Cantabrana et al. (2019)	C	25	Q	27
Napal et al. (2018)	C	44	TP	24
Cózar-Gutiérrez et al. (2016)	Q	162	Q	18
Moreno et al. (2018)	Q	104	Q	17
Gutiérrez & Serrano (2016)	Q	134	Q	17
Domingo-Coscollola et al. (2019)	M	484	Q, DG and IN	16
Fernández-de-la-Iglesia et al. (2020)	Q	526	C	16
Castillejos et al. (2016)	M	62	Q and IN	15
Girón-Escudero et al. (2019)	M	117	Q	13
Gutiérrez & Cabero (2016)	Q	2038	Q	13
Gallego-Arrufat et al. (2019)	Q	317	Q	12
Xu et al. (2019)	Q	905	Q	12
Karaduman (2017)	Q	65	Q	11
Liesa et al. (2016)	Q	960	Q	*8*
Flores-Lueg & Roig Vila (2016)	C	54	FG	7
Silva et al. (2019)	C	568	Q	6
Björk et al. (2020)	Q	1244	Q	5
Ogunlade et al. (2013)	Q	150	Q	5
Karakoyun & Lindberg (2020)	Q	197	Q	4
Gómez-del-Castillo & Gutiérrez-Castillo (2015)	C	34	Q and FG	3
Aristizabal & Cruz (2018)	C	171	RD, T, R, PA and SA	3
Torres-Hernández et al. (2019)	C	154	I, Q	2
Çebi & Reisoğlu (2020)	C	518	Q	1
Usart et al. (2021)	Q	144	Q	1
Roll & Ifenthaler (2021)	Q	222	Q	0
Suárez-Guerrero et al. (2020)	Q	9469	Q	0
Moreno-Guerrero et al. (2020)	Q	153	Q	0
Mercader and Gairin (2021)	Q	337	Q	0

*Instruments = Questionnaire (C), Interview (IN), Discussion groups (DG), Focus group (FG), Intervention (I), Reflective diary (RD), Peer assessment (PA), Self-assessment (SA), Tasks (T), Training program (TP), Rubrics (R) and Lesson plans (LP)

When classified by study methodology, 62.5% of the articles chosen use a quantitative focus, 25% are qualitative studies, and 12.12% use a mixed methodology. The prevalence of quantitative studies is a determining factor for achieving a total sample of 20.068 participants. Studies with large samples were performed from a quantitative perspective, whereas studies with small samples tended to use qualitative approaches.

As to the instruments and techniques for data collection, the quantitative studies use survey technique with questionnaires. In 9 studies, the instruments were constructed ad hoc. Seven referred to indicators from the European digital competence framework. Three were adapted from other authors, and the rest were construed from different countries’ national frameworks. Two used the Digital Teaching Competence Framework of the National Institute for Educational Technology and Spanish Education (INTEF, 2017). The qualitative studies used techniques and strategies belonging to this methodology, such as interviews (semi-structured and in-depth), discussion groups, focus groups, training programs, tasks, lesson design, narratives and assessment techniques and instruments, such as rubrics, peer assessment and self-assessment.

- RQ3. What competence levels do preservice teachers have in research on digital security?

Among studies that treat competence levels, Napal et al. (2018), Björk et al. (2020) and Çebi and Reisoğlu (2020) find that preservice teachers show a high/advanced competence level in the area of security. According to the studies by Moreno et al. (2018), Torres-Hernández et al. (2019), Pascual et al. (2019), Grande de Prado et al. (2020) and Gabarda et al. (2017), preservice teachers have a medium level. This last group of studies finds that the lowest competence levels occur in management of digital identity, copyright and licenses, and innovation and creative technology use. The results of Xu et al. (2019) show low levels among preservice teachers in issues related to digital security, health and digital wellbeing.

Analysing competence levels for the variables of age and gender, Usart et al. (2021) find that preservice teachers’ age correlates very highly with the ethical dimension and with security.

- RQ4. What indicators are found in studies of preservice teachers’ digital competences that enable assessment of digital security?

The analysis performed shows that 48% of studies include items or dimensions. All of the items are linked to the area of security in general or specifically to protection of devices, protection of personal and digital data, protection of the environment, or protection of health. Studies by Gallego-Arrufat et al. (2019), Castillejos et al. (2016), Björk et al. (2020) and Çebi and Reisoğlu (2020) explore and deepen knowledge of digital security. Gallego-Arrufat et al. (2019) designed a 59-item instrument with 7 dimensions related to the area of digital teaching competence in security: *protection of personal data, management of digital identity, netiquette, interaction through technology, sharing of information and digital content, protection of health and bullying on social networks, Internet and cell phone technology.* Castillejos et al. (2016) focus their study on analysing security practices, with 29 items related to 4 competences: *protection of devices, protection of personal data, protection of health and protection of the environment.* Björk et al. (2020) employ a questionnaire on responsible technology use, including 3 items that focus on the *dimension of privacy* to analyse what competences preservice teachers must acquire to use technologies in their own learning for teaching in the future. In their 5-dimension questionnaire on digital competences, Çebi and Reisoğlu (2020) include the dimension of *Safety*.

Other studies also include items related to digital security, as well as other dimensions of digital competence. In a single item related to *Information security*, located in the section *Storage and recovery of information, data and digital content*, Moreno-Guerrero et al. (2020) observe that older participants show a higher competence level in creating back-up copies. Fernández-de-la-Iglesia et al. (2020) include items on limitations for use based on criteria related to intellectual property, administration of the computer’s resources, and use and management of security software. Suárez-Guerrero et al. (2020) include two items in the factor on the *dimension of digital citizenship*: Demonstrating personal responsibility for life-long learning using ICTs and Understanding digital etiquette (netiquette) by conducting responsible social interaction related to information use and ICTs. Cózar-Gutiérrez et al. (2016) include 7 items related to *security* and ask the teachers in training their opinions about the ICT tools. They do not consider whether the tools students use provide security that protects the students’ privacy. Xu et al. (2019) include 3 items on interest in communicating and protecting one’s rights within the dimension of *Assertiveness* in interpersonal communication competence. This dimension relates digital citizenship to digital security, health, digital wellbeing, and digital rights and responsibilities. Gutiérrez and Cabero (2016) include 6 items on behavioural norms for technology use in the category *Digital citizenship* and *search for and treatment of information,* 7 items on behavioural norms for use of technologies that treat ethical commitment in the use of digital information and ICTs; promotion and practice of secure, legal, responsible use of information and ICTs; and responsible attitude toward learning with technologies.

Napal et al. (2018) design an instrument with items related to the four digital security competences. They also describe and relate *security* to *protection of information and personal data, protection of digital identity, security measures* and *secure responsible use* to other subcompetences, such as *protection of health and the environment.* Zempoalteca et al. (2017) analyse students’ academic practices with two items on security: information sharing through social networks and sending documents by email. Gutiérrez and Serrano (2016) include items on *protection and updating of devices, personal privacy and privacy* of others*, care for health* and *negative effects on the environment due to indiscriminate technology use*.

We also find publications in which the area of security appears as a dimension in the study. Moreno et al. (2018) faithfully reproduce the dimension of security with the four competences and indicators from the DigCompEdu Framework. Castillejos et al. (2016) include 29 items with *knowledge and capabilities to perform activities related to competence in security*. Ogunlade et al. (2013) include items that explore *downloads from Internet, browsing on pornography websites, participation in fraud and knowledge of norms for online behaviour*. Domingo-Coscollola et al. (2019) describe the dimension of analysis *Digital ethics and civility* using the following descriptors: protection of fundamental rights to personal privacy and to one’s image; responsible, secure, healthy use of digital technologies; promotion of access to resources while respecting intellectual property; fostering digital inclusion and fostering construction of an appropriate digital identity. Silva et al. (2019) include an 8-item dimension to assess *ethical, legal and security issues*. Their assessment achieves higher point-values for preservice teachers in the two countries studied. Other studies include dimensions directly related to the area of *Security* in the DigCompEdu Framework. Usart et al. (2021) include the dimension *Legal, ethical, and security issues* in their instrument. Lázaro-Cantabrana et al. (2019) design an instrument to assess digital competence in which one dimension incorporates issues related to *ethics and security*. Mercader and Gairín (2021) propose the dimension *Ethical and secure social interaction*, with indicators such as ethics and security, digital identity of the school and digital identity and presence, as well as creation and use of open source materials.

Other scholars analyse and approach security from different perspectives, focusing on Teacher Education. Karakoyun and Lindberg (2020) identify questions of *digital citizenship and critical thinking* as important topics related to everyday life in the twenty-first century and skills that preservice teachers should have. Gallego-Arrufat et al. (2019) design an educational intervention in the form of a partially-classroom-based workshop for preservice teachers that revolves around *digital security and privacy on Internet*, with activities and tools that develop and reflect on different problems associated with the topic. Aristizabal and Cruz (2018) tackle improvement of this area of competence starting from student participation through self-assessment of the five areas of DigCompEdu. These areas include security and identifying what was learned, what has improved and self-assessment. The participants analyse security policies and incorporate them into their blogs. Karaduman (2017) incorporates characteristics and *digital citizenship competences related to the area of security* as ethical values and attitudes, respect, responsibility, rights and obligations. In the study by Shin (2015), the participants *design lessons with online materials and assess them*, and observe that the participants barely consider the quality of the materials, copyright and security-related goals when they do so. Prendes-Espinosa et al. (2010) include items on *identification of email spam, ability to assess authorship and reliability of information taken from Internet, and knowledge of rights and responsibilities* as users of the university computer network. Gómez-del-Castillo and Gutiérrez-Castillo (2015) include the *dimension of digital citizenship*, exploring questions related to secure, legal, responsible information use.

- RQ5. What opportunities for improvement in teachers’ digital competence in security appear in the studies?

The studies included in this review relate issues of digital security to ethical, critical and civic issues. Security is an emerging dimension that must be included in Teacher Education. It is closely related to digital care of minors; responsible, secure, healthy use of digital technologies; protection of the right to personal privacy and to one’s image; and promotion of access to and use of resources while respecting intellectual property (Domingo-Coscollola et al., 2019). It involves applying measures to protect digital devices and content, taking measures to ensure security and privacy in the online environment (Çebi & Reisoğlu, 2020), knowing about privacy, handling cyberbullying, assessing digital content (Björk et al., 2020), recognizing dangers concerning security on websites and integrating topics such as secure Internet, copyright and citation into courses (Karaduman, 2017).

Digital security is currently a question of great importance. Teacher Education must attend to this question to strengthen the critical dimension of technology use during acquisition of digital competences and to review its presence in current study programs (Xu et al., 2019; Moreno et al., 2018). Initial teacher training is the ideal time to change actions and practices to generate open attitudes favourable both to educational innovation with ICTs and to increasingly crucial digital literacy, as well as to effective communication of information in an ethical and legal way to build knowledge (Liesa et al., 2016). It is thus necessary to make a qualitative leap from common technical skill to competent, critical technology use (Suárez-Guerrero et al., 2020). In this vein, Flores-Lueg and Roig-Vila (2016) hold that the society in which preservice teachers and children are living requires them to use technologies, and this demand includes generation of deeper changes in teaching practices, changes that go beyond projecting slides or updating technological equipment. Education must include control of the environment, immediacy and management of interaction, combined with information on the dimensions Protect Yourself (PY) Protect Others (PO) for citizens (Shun et al., 2018). According to Björk et al. (2020), preservice teachers’ digital competence and ability to use ICTs responsibly is an essential part of teacher education programs, and students must know these perspectives to better face the challenges they will encounter in the classroom when they begin work as novice teachers in the field of education. Citizens of the preservice demand knowledgeable individuals, properly educated and trained for precise, effective use of ICTs in the personal, academic, work-related and social spheres (Cózar-Gutiérrez et al., 2016). The university must therefore foster education in and use of ICTs that focus on competences, abilities and skills for training in educational and social use. Such action can foster attitudes that enable teachers subsequently to transfer this competence to their students (Gutiérrez & Cabero, 2016). To achieve this goal, we propose extracurricular activities, projects and symposia on digital citizenship (Karaduman, 2017), seminars, courses (Björk et al., 2020) and inclusion of training programs and practical activities in teacher education programs. This training must be oriented to issues such as how to design activities that support development of this knowledge and skills (Çebi & Reisoğlu, 2020).

## Conclusions

The goals of this review were to gather the scholarly production and analyse the studies on indicators assessing the area of security in DigCompEdu.

The last three-year period of the decade analysed shows high productivity of publications, with significant increase starting in 2016. This year coincides with the publication of the European Digital Competence Framework for Citizens, which provides a guide for the creation of reference frameworks in various parts of the world. In 2019 and 2020, substantial growth occurred, coinciding with the onset of the COVID-19 pandemic, during which technology use was a determining factor in nearly all education systems worldwide.

Although this review focused on the field of digital security, the keyword search showed that “Safety/e-Safety” was the least frequent keyword, compared to others such as *Digital Competences* or *Teacher Training*. The emergence and use of these keywords in scholarly production in education research on digital competence are clearly still in the early stages, as are articles that focus study solely on this topic.

By participants’ country of origin, the review shows interest in the topic in the Americas, Asia and Europe, with a higher index of scholarly production in Europe.

It is worth noting the predominance of quantitative over qualitative and mixed studies, with the self-assessment survey as the instrument most frequently used. The review also finds studies that treat this competence area from a more holistic perspective, however, and that are more closely related to educational and methodological issues for preservice teachers. This is the case for interventions and education programs for preservice teachers. The foregoing enables alternatives for assessment and education on this topic that do not base assessment of digital competence in security only on self-perceptions of the participants, who generally assess their competence as higher than it really is (Maderick et al., 2016). The foregoing also enables us to imagine an opportunity to tackle the Teacher Training needed and proposed in some studies based on the indicators included in the studies reviewed here, as well as the use of varied techniques and instruments for intervention and assessment.

It is central to stress that numerous studies of digital teaching competence focus on information literacy and few on issues of digital security, competences more closely linked to legal, ethical and critical questions. In the review, only a very few studies relate health and care for the environment, two topics that in Europe are expressly related to the area of digital competence in security.

On the other hand, the review demonstrates that digital security requires responsible knowledge, practice and attitudes to Internet and technology use. In the results on RQ5, studies with training actions or programs acquire value in this context, with training programs that work with methods for teaching how to design lessons while respecting standards of authorship of resources, data protection and privacy. These strategies are useful for improving training and increasing awareness of the risks and problems that are not usually recognized until the risk has become a problem that affects or impacts classrooms and society.

Further, as to assessment, practices noted in these studies are beneficial in that they enable assessment of digital competences based on evidence from conducting activities related to digital security and how one should teach and act in classrooms.

The studies of digital competence that include some item, dimension or mention of the area of security competence propose the need for and importance of educating pre-service teachers on this topic. One cannot merely trust these educators’ condition as digital natives, as this condition does not guarantee the competences needed for teaching to orient students and make them aware of the importance of digital education. The results of RQ3 on the self-assessments in this field demonstrate this need; none of the studies shows high levels of competence in security. Some studies, in contrast, indicate that the skills that score lowest include those linked to security, such as identity management, copyright and licenses (Rodríguez et al., 2020). Others recognize that less improvement has occurred in the area of security (Gutiérrez & Serrano, 2016) and that this is the area that still presents substantial difficulties (Napal et al., 2018).

From our perspective, the indicators and results presented constitute the starting point for proposing the need to analyse the study programs and curricular content composing the different subjects that focus on education and technologies in universities. The goal of this analysis is to detect possible inequalities in the relationship to the dimensions that compose digital competence. As Zempoalteca et al. (2017) indicate, the goal is to have a balanced impact on the training of preservice teachers. In education for security, it is not enough for preservice teachers to be aware of the existence of problems such as cyberbullying, sexting, breaches of online privacy or risks of one’s reputation. They need social, didactic and methodological competences to design effective programs, to know how to face these risks and to have sufficient knowledge to explore the online reality responsibly and securely.

The curricular content related to the digital security studied here and/or to transversal treatment during the initial teacher training identified here includes sufficient protection of devices from security breaches, which are occurring constantly; protection of personal data and privacy; management of digital identity in the virtual world, search for and use of Internet images with copyright screening; use of free software programs; respect for online communication norms; and ethical and responsible technology use. We believe that such measures will in the near future enable more solid initial training in matters of security to face problems that arise in everyday life when one uses or interacts with a technological artifact or Internet. Such initiatives are important because the effects of security go beyond instrumental issues; their consequences have social and psychological implications that concern citizens’ digital education.

### Weaknesses of the study

From the conceptual point of view, one of the most significant limitations has been the scarcity of studies that treat only the topic of digital security with focus on training for teaching in any of its stages. This finding led us to restrict the publication search to a period of only 10 years. This study thus covers a very limited period of time relative to the trajectory of ICTs in initial teacher training.

At methodological level, the identification of useful studies in this systematic review involves using a very generic search chain on digital competence and purging all articles that did not fit the topic and questions. This method led the authors to perform numerous readjustments of the criteria of inclusion to reach the criteria we considered the most suitable for this study.

The studies included and their contexts to some extent limit our ability to provide a broader panorama of initial teacher training in universities in North American, Asian and African contexts that attend to the problems stemming from technology use and that may thus present models to train faculty on this topic. The conclusions cannot, therefore, be generalized. In addition to the limitation that research on the topic of this review is in the early stages, we find that not all studies provide complete information on the indicators they use, limiting our ability to provide more in-depth of analysis of them.

### Recommendations for future research

We propose the following lines of research:

- First, to research the way institutions that educate preservice teachers have addressed or resolved the educational gaps in matters of digital security of preservice teachers and of their educators before and after the pandemic.
- Second, to explore in-service and pre-service teaching practices related to risks in data protection, authorship and digital rights by seeking, selecting and sharing information.

Ultimately to conduct studies of digital competence in matters of security that enable identification of the didactic, technical and methodological needs and qualifications of preservice teachers, both in educational practice and for future practice in the classroom.

## Data Availability

Not applicable.
